# Pharmacologic Inhibition of Erythrocyte Ferroportin Expression Exacerbates *Plasmodium* Infection

**DOI:** 10.3390/microorganisms13081859

**Published:** 2025-08-08

**Authors:** Sareh Zeydabadinejad, Benjamin Frederick Theis, Jun Sung Park, Amira F. Gohara, Matam Vijay-Kumar, Beng San Yeoh, Piu Saha

**Affiliations:** 1 Center for Hypertension and Precision Medicine, Department of Physiology and Pharmacology, University of Toledo College of Medicine and Life Sciences, Toledo, OH 43614, USA; sareh.zeydabadinejad@utoledo.edu (S.Z.); benjamin.theis@rockets.utoledo.edu (B.F.T.); junsung.park@rockets.utoledo.edu (J.S.P.); matamvijay.kumar@utoledo.edu (M.V.-K.); bengsan.yeoh@utoledo.edu (B.S.Y.); 2 Department of Pathology, University of Toledo Medical Center, Toledo, OH 43614, USA; amira.gohara@utoledo.edu

**Keywords:** *P. yoelii*, erythropoietin, VIT-2763, vamifeport, reticulocytes, iron homeostasis, malaria, anemia, transferrin, RBC

## Abstract

*Plasmodium* parasites rely on host iron for survival and replication, making host iron availability a critical determinant of malaria pathogenesis. Central to iron homeostasis is the hepcidin–ferroportin regulatory axis, where hepcidin suppresses iron export by inducing ferroportin degradation, thus modulating systemic and cellular iron availability. In the *Plasmodium* infection model (*P. yoelii*), we observed a significant downregulation of hepatic *hepcidin* expression, accompanied by an increase in hepatic *ferroportin* expression. On the contrary, RBC-ferroportin protein level was notably suppressed upon *P. yoelii* infection. Given these findings, we aim to investigate the role of a ferroportin inhibitor in *Plasmodium* infection. In a *P. yoelii* mouse model, treatment with an oral ferroportin inhibitor, VIT-2763 (Vamifeport) increased parasitemia, accompanied by increased levels of pro-inflammatory cytokines, erythropoietin, and liver injury markers. In *P. yoelii* infected mice, VIT-2763 treatment suppressed *hepcidin* expression and increased *ferroportin* expression in hepatocytes, while reducing ferroportin protein levels in RBCs. VIT-2763 mediated exacerbation of *P. yoelii* infection reveals the tissue-specific regulation of ferroportin in hepatocytes and RBCs, underscoring the therapeutic potential of modulating the hepcidin–ferroportin axis as an intervention strategy in malaria.

## 1. Introduction

Malaria is a debilitating disease caused by the protozoan parasite, *Plasmodium* spp. It remains a global health challenge, particularly in tropical and subtropical regions, where *Plasmodium* infections led to over 200 million cases and nearly half a million deaths annually [1,2,3]. Various studies have shown that malaria and iron have an intricate relationship, as iron is the principal micronutrient required for the proliferation of *Plasmodium* parasites [4,5,6]. Hemoglobin breakdown by the parasite releases substantial amounts of free heme, which is highly toxic to the parasite due to its potential to disrupt membranes, induce lipid peroxidation, and ultimately lead to parasite death. To counteract these harmful effects, the parasite converts free heme into an insoluble, inert crystalline form known as hemozoin. Hemozoin’s unique biophysical properties also contribute to immune activation and pathogenesis, highlighting its significance as both a parasite’s detoxification product and a bioactive molecule influencing the host response [7,8]. Additionally, clinical studies have demonstrated that iron supplementation increases risks of *Plasmodium* infection and disease severity [9] whereas iron deficiency appears to confer protection against malaria. Understanding the interplay between host iron metabolism and malaria pathogenesis has therefore emerged as a promising area for therapeutic intervention [4,6,9].

The hepcidin–ferroportin (FPN) axis plays a pivotal role in regulation iron homeostasis in mammals. Hepcidin, a liver-derived peptide hormone, regulates systemic iron levels by binding to FPN, the sole known cellular iron exporter [6]. Hepcidin binding triggers FPN internalization and degradation, thus reducing iron export from macrophages, hepatocytes, and enterocytes. This lowers the levels of serum iron and increases intracellular iron stores. While this pathway is essential for maintaining iron balance under physiological conditions, its dysregulation during infections or inflammation has significant consequences [6,10]. For instance, elevated hepcidin levels during *Plasmodium* infection may limit iron availability to pathogens but also exacerbate anemia, a common complication in malaria. Yet, excessive FPN activity can also reduce intracellular iron, potentially depriving pathogens of the iron required for their survival [4,9,11].

Previous studies have highlighted the critical role of the hepcidin-FPN axis in regulating iron availability within red blood cells (RBC), influencing *Plasmodium* infection outcomes. This is achieved via FPN facilitating iron egress from RBC and thereby limiting the iron supply to the parasite. However, elevated iron levels or increased hepcidin expression reduce FPN activity, leading to iron retention within cells, promoting parasite growth, and worsening infection severity [11,12]. Interestingly, a FPN mutation (Q248H) common in African populations confers resistance to hepcidin regulation, potentially offering protection against malaria [13]. Herein, in our infection study, we noticed a significant increase in erythropoietin (EPO) levels in *P. yoelii* infected mice, along with downregulation of hepatic *hepcidin* expression and a marked upregulation of *Fpn* expression. The expression of FPN on RBCs, however, was significantly suppressed during *P. yoelii* infection.

Building on these insights, we explored the use of VIT-2763 (Vamifeport, VIT), a novel oral FPN inhibitor currently under development for the treatment of hemoglobinopathies such as sickle cell anemia and β-thalassemia [14,15,16,17,18], and is undergoing phase 1 trials in healthy volunteers [19]. Herein we observed, VIT treatment (orally, 30 mg/kg bw, 7 days) led to an increased parasite burden along with a substantial increase in systemic pro-inflammatory mediators, lipocalin-2, serum amyloid A, and liver injury markers. Moreover, compared to untreated mice, VIT treatment further increased both circulatory Ter119^+^ CD71^+^ reticulocytes and serum erythropoietin (EPO) levels, confirming greater dyserythropoesis in VIT-treated *P. yoelii* infected mice. Additionally, VIT-treated mice exhibited a more pronounced downregulation of hepatic *hepcidin* following *P. yoelii* infection. Concurrently, a greater upregulation of hepatic *Fpn* and a more noticeable suppression of FPN in RBC (RBC-FPN) were observed in the VIT-treated group. Together, these results demonstrated that disrupting the hepcidin-FPN axis can exacerbate the severity of *P. yoelii* infection, and highlighting the importance of iron metabolism, in malaria pathogenesis. Overall, our results provide a compelling insight into the role of iron regulation in malaria, especially site-specific ferroportin expression (hepatic vs. RBC) during malaria, highlighting hepcidin-FPN axis as a potential therapeutic target.

## 2. Materials and Methods

### 2.1. Mice

C57BL/6J wild-type mice (Stock # 000664) were obtained from Jackson Laboratory and bred in-house at the Department of Laboratory Animal Resources, University of Toledo College of Medicine and Life Sciences. The mice were housed at 23 °C with 12 h light/dark cycles, pathogen-free air, with food and water available ad libitum. For all experiments, age- and sex-matched mice (both males and females) were used. All animal handling procedures adhered to Institutional Animal Care and Use Committee (IACUC)-approved protocols.

### 2.2. Plasmodium yoelii (P. yoelii) Infection and Parasitemia Quantification

The *Plasmodium yoelii* subs *P. yoelii* strain 17XNL: PyGFP (Catalog No. MRA-817) [20], which stably expresses green fluorescent protein (GFP), was sourced from BEI Resources and stored as frozen stocks of parasitized red blood cells (RBCs). Mice were infected via bilateral intraperitoneal (*i.p.*) injection of 1 × 10^5^ * P. yoelii*-parasitized RBCs in 100 µL of sterile PBS on each side of the body. Parasitemia was evaluated by flow cytometry, defined as the percentage of GFP-positive RBCs in whole blood collected from the tails of infected mice. At the end of the experiment, mice were euthanized by CO_2_ inhalation, and serum along with organs were collected and stored at −80 °C for further analysis.

### 2.3. Administration of VIT-2763

Stock solution (10 mg/mL) of VIT-2763 (MedChemExpress, Monmouth Junction, NJ, USA) was prepared in DNase- and RNase-free ultra-pure distilled water and administered to mice via oral gavage on 7 consecutive days at a dose of 30 mg/kg B.W. In the *P. yoelii*-infected group, the first dose of VIT was administered 18 h post-infection. Control mice received an equivalent volume of DNase- and RNase-free ultra-pure distilled water at the corresponding time points.

### 2.4. Flow Cytometric Analysis of Reticulocytes

Approximately 2 µL blood was collected via tail nick and diluted in 1 mL of sterile PBS (pH 7.0). After centrifugation at 1200 rpm for 5 min, the supernatant was discarded, and the cells were resuspended in PBS containing 0.2% FBS. The washing process was repeated twice, followed by resuspension in 100 µL of PBS with 0.2% FBS. The cells were then stained with fluorophore-conjugated anti-mouse monoclonal antibodies (Ter119-PE, CD71-APC, BD Biosciences, Franklin Lakes, NJ, USA) and incubated for 40 min at room temperature in the dark. After a final wash, the stained cells were analyzed using the Accuri C6 flow cytometer (BD Biosciences) and BD Accuri C6 Plus, Software (version 1.0.34.1).

### 2.5. Serum Collection

Baseline serum samples were collected two weeks prior to the start of the experiment and collected again at the time of euthanasia. Blood samples were collected into BD Microtainer tubes (BD Biosciences). The blood was centrifuged at 10,000 rpm for 10 min, and hemolysis-free serum was collected and stored at −80 °C until further analysis.

### 2.6. Preparation of Red Blood Cell (RBC) Ghosts

RBC membranes (ghost) were prepared as described by Hanahan and Ekholm [21]. Blood was first collected into EDTA-coated tubes (Sarstedt Inc., Newton, NC, USA) to prevent clotting. The blood was centrifuged at 1000× *g* for 10 min at 4 °C to separate the plasma from the RBCs. All subsequent steps were performed on ice to maintain sample integrity. The RBCs were then washed three times with 1 mL of 0.172 M Tris (isotonic buffer, pH 7.6) by resuspending them in the buffer and centrifuging at 1000× *g* for 10 min at 4 °C after each wash. Once cleaned, the RBCs were gently resuspended in 1 mL of 0.011 M Tris (lysis buffer, pH 7.6). The mixture was centrifuged at 15,000× *g* for 15 min at 4 °C to pellet the RBC membranes (ghosts) and remove hemoglobin and other cytoplasmic components. This step was repeated until the supernatant became clear, and a nearly clear pellet was obtained. The final supernatant was discarded, and the ghosts were stored at −80 °C until analysis for protein estimation and immunoblot.

### 2.7. Hematological Analyses

Blood samples, collected at the time of euthanasia, were drawn into EDTA-coated microtubes for complete blood count (CBC) analysis using the automated VetScan HM5 Hematology Analyzer (Abaxis HM5C & VS2, Allied Analytic, Tampa, FL, USA). Key hematological parameters measured included white blood cell (WBC) and red blood cell (RBC) counts, hemoglobin concentration, hematocrit, mean corpuscular volume (MCV), mean corpuscular hemoglobin (MCH), and mean corpuscular hemoglobin concentration (MCHC).

### 2.8. Estimation of Serum Iron

Non-heme iron was estimated in serum after acid digestion as outlined in Torrance and Bothwell [22]. Briefly, serum samples (50 μL) were incubated in acid solution (3M HCl containing 10% trichloroacetic acid) for 15 min. Next, the digested samples were centrifuged and 30 μL of the supernatant was transferred to a 96-well microplate (Corning, Corning, NY, USA). The working chromogen reagent was prepared freshly on the day of assay from chromogen reagent stock (0.1% bathophenanthroline sulphonate and 1% thioglycolic acid). Next, 30 μL of working chromogen reagent (1 volume of chromogen reagent stock, 5 volumes of saturated sodium acetate and 5 volumes of dd H_2_O) were added to the samples and incubated at RT for 10 min. Absorbance was measured at 562 nm. A standard curve was generated using the iron atomic absorption (AA) standard, and iron concentration was determined by extrapolating the absorbance of unknown samples on the standard curve.

### 2.9. Tissue Iron Estimation

Non-heme iron was estimated in liver and spleen after acid digestion as outlined in Torrance and Bothwell [22]. Briefly, tissue samples (50 mg/mL) were digested in acid solution (3M HCl containing 10% trichloroacetic acid) and incubated at 65 °C for 24 h. Next, the digested samples were centrifuged and 25 μL of the supernatant was transferred to a 96-well microplate (Corning). The working chromogen reagent was prepared freshly on the day of assay from chromogen reagent stock (0.1% bathophenanthroline sulphonate and 1% thioglycolic acid). Next, 250 μL of working chromogen reagent (1 volume of chromogen reagent stock, 5 volumes of saturated sodium acetate and 5 volumes of double deionized water) was added to the samples and incubated at RT for 10 min. Absorbance was measured at 562 nm. A standard curve was generated using the iron atomic absorption (AA) standard and iron concentration was determined by extrapolating absorbance of unknown samples on the standard curve.

### 2.10. Measurement of Liver Injury Markers

Serum total bile acids (TBA) were measured using the Diazyme Total Bile Acids Assay Kit (Diazyme, Gregg Ct. Poway, CA, USA, Cat # DZ042A) following the manufacturer’s instructions. Serum total cholesterol, aspartate transaminase (AST), and alanine aminotransferase (ALT) levels were measured using Randox assay kits (Kearneysville, VA, USA, Cat # CH200, AL146, and AS147, respectively) according to the manufacturer’s protocols.

### 2.11. Serum ELISA

Serum lipocalin 2 (LCN2, Cat # DY1857), serum amyloid A (SAA, Cat # DY2948), and erythropoietin (EPO, Cat # DY959) were quantified in hemolysis-free serum using DuoSet ELISA kits from R&D Systems (McKinley Place NE, Minneapolis, MN, USA).

### 2.12. Quantitative Real-Time PCR Analysis

Total RNA was extracted from liver samples using TRIzol™ Reagent (Invitrogen, Waltham, MA, USA), following the manufacturer’s instructions. RNA concentration and purity were assessed using a NanoDrop One spectrophotometer (Thermo Fisher Scientific, Waltham, MA, USA). cDNA was synthesized from 2 µg of total RNA using the qScript^®^ cDNA SuperMix (Quantabio, Beverly, MA, USA) according to the manufacturer’s protocol. Quantitative real-time PCR (qRT-PCR) was performed on an Applied Biosystems StepOnePlus Real-Time PCR System using gene-specific primers for *hepcidin*, *ferroportin*, and *transferrin*. *18S rRNA* was used as the housekeeping gene for normalization. The following primers were used to assess gene expression: *hepcidin* forward primer: 5′-AGAAAGCAGGGCAGACATTG-3′ and reverse primer: 5′-CACTGGGAATTGTTACAGCATT-3′; *ferroportin (Fpn)* forward primer: 5′-TTGTTGTTGTGGCAGGAGAA-3′ and reverse primer: 5′-AGCTGGTCAATCCTTCTAATGG-3′ [23]; *transferrin* forward primer:5′-TGTAGCCTTTGTGAAACACCAGA-3′ and reverse primer: 5′-TCGGCAGGGTTCTTTCCTT-3′ [24]; *18S* forward primer: 5′-GTTCAGCCACCCGAGATTGA-3′ and reverse primer: 5′-TGTGTACAAAGGGCAGGGAC-3′ (Cambridge University Press, Cambridge, UK). Relative gene expression levels were determined by the 2^^(−ΔΔCT)^ method.

### 2.13. Immunoblot Analysis for RBC Ferroportin

RBC ghosts (RBC membrane) were homogenized in RIPA buffer containing protease and phosphatase inhibitors. After centrifugation at 13,000× *g* for 15 min at 4 °C, the supernatant was collected and protein concentration measured. 25.0 μg of protein/lane were separated by SDS-PAGE and electroblotted onto PVDF membranes. The membranes were blocked in 5% non-fat milk containing 20 mM Tris-HCl, pH 7.6, 137 mM NaCl (TBS) for 2 h at room temperature, and then incubated with rabbit anti-mouse Fpn1 (1:5000) (Alpha Diagnostic International, San Antonio, TX, USA) for overnight at 4 °C. After washing with 20 mM Tris-HCl, pH 7.6, 137 mM NaCl, 0.1% Tween-20 (TBS-T) three times, the membranes were incubated in anti-rabbit IgG, horseradish peroxidase-linked antibody (1:2000) (Cell Signaling, Danvers, MA, USA, Cat #7074) for 1 h at room temperature. Immunoreactive proteins were detected by using the enhanced chemiluminescence method (Pierce™ ECL Western Blotting Substrate, SW Lakewood, WA, USA, Cat # 32209). After protein transfer, membranes were stained with Ponceau S to confirm uniform protein loading and transfer across all samples. Band intensities corresponding to FPN were quantified using ImageJ.JS. software (NIH), based on the integrated density of the bands. Ponceau S staining is shown as loading control.

### 2.14. Histological Analysis of Liver and Spleen Sections

Liver and spleen samples from uninfected and infected mice were fixed in 10% neutral buffered formalin (NBF), embedded in paraffin, sectioned (2 µm), and stained with Hematoxylin and Eosin (H&E). Histological images were obtained using the VS120 Virtual Slide Microscope (Olympus, Tokyo, Japan) and visualized with OlyVIA 2.9 software. Histological scoring was conducted by a board-certified pathologist in a blinded fashion.

### 2.15. Statistical Analysis

Data are presented as mean ± SEM. Statistical significance between two groups was assessed using a two-tailed, unpaired Student’s *t*-test (*p* < 0.05 was considered significant). For comparisons among three or more groups, one-way ANOVA with pairwise multiple comparisons was performed. All analyses were conducted using GraphPad Prism 9.0 (GraphPad Software, Inc., La Jolla, CA, USA).

## 3. Results

### 3.1. P. yoelii Infection Suppressed Hepatic Hepcidin Expression and Increased Ferroportin (Fpn) Expression

Hepcidin, the master regulator of systemic iron homeostasis, plays a complex and critical role during malaria by restricting iron availability and influencing disease susceptibility [25,26,27]. In the context of *P. yoelii* infection, *hepcidin* expression may be either upregulated or downregulated based on the stage and severity of infection [28], which subsequently alters the expression of *Fpn*, the sole known cellular iron exporter.

**Figure 1:**
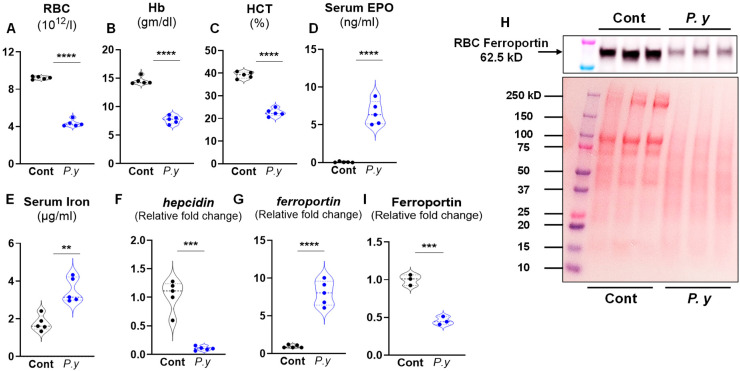
***P. yoelii* infection induced anemia and disrupted the iron homeostasis.** Wild type (WT) mice (10-week-old males) were divided into two groups (*n* = 5/group). One group was infected with *P. yoelii*, and the control group received a PBS injection. Mice were euthanized 12 days post-infection (*p.i.*). Blood samples from control and *P. yoelii* infected mice were collected (EDTA tubes) for complete blood count (CBC; via VetScan hematology analyzer) analysis. Results for: (**A**) Red blood cells (RBC); (**B**) Hemoglobin (Hb); (**C**) Hematocrit (HCT, the volume percentage of RBC in blood). Serum samples were analyzed for (**D**) erythropoietin (EPO) measured by ELISA. (**E**) Seum iron. Hepatic mRNA expression of (**F**) *hepcidin*, (**G**) *ferroportin. 18s* used as the housekeeping gene. (**H**) Immunoblot for Ferroportin in RBC membrane (*n* = 3/group). Each lane represents an RBC membrane fraction isolated from a single mouse. Ponceau S staining is shown as loading control. (**I**) Densitometric analysis of immunoblot by ImageJ.JS. Data represented as mean ± SEM. ** *p* < 0.01, *** *p* < 0.001, **** *p* < 0.0001.

Iron dysmetabolism and anemia play a key role in the progression of *Plasmodium* infection, influencing both host defense and parasite survival [6]. To investigate iron regulation in *P. yoelii*-infected mice, we first confirmed the presence of anemia at day 12 *p.i.*, as evidenced by significant reductions in RBC count, hemoglobin concentrations, and hematocrit (Figure 1A–C). Impaired erythropoiesis was further supported by a marked increase in serum erythropoietin (EPO) levels (Figure 1D). As expected, we noticed that serum iron levels were significantly elevated in *P. yoelii* infected mice (Figure 1E). Hepatic *hepcidin* expression was significantly suppressed on day 12 *p.i.* (Figure 1F), while hepatic *Fpn* expression was upregulated (Figure 1G). More significantly, immunoblot results demonstrated that FPN levels in RBCs decreased (Figure 1H,I) on day 12 *p.i.* These findings confirm that *P. yoelii* infection disrupts the hepcidin-FPN axis, contributing to dysregulated iron homeostasis during infection.

### 3.2. Inhibition of FPN by VIT Lowers the Circulating Iron and Enhances Liver Iron Storage

Given the increased *Fpn* expression in *P. yoelii*-infected mice, we hypothesized that such increase might either exacerbate infection or present a compensatory response to inflammation. To investigate this, we pharmacologically targeted FPN using the oral ferroportin inhibitor VIT-2763 (VIT). First, we evaluated the impact of VIT on hematological, immunological and iron-related parameters in healthy WT mice. Orally administered VIT (single dose of 30 mg/kg bw of VIT on 7 consecutive days) did not cause any significant changes in body and/or organ weights (Supplementary Figure S1A–D). Hematological analysis revealed a moderate increase in RBC numbers (Supplementary Figure S1E), while there was no difference in other hematological parameters including hemoglobin (Hb), hematocrit (HCT), mean corpuscular volume (MCV), mean corpuscular hemoglobin (MCH), mean corpuscular hemoglobin concentration (MCHC) and platelets (Supplementary Figure S1F–K). Although immune cell parameters such as WBC, lymphocyte and monocyte counts remained unchanged (Supplementary Figure S1L–N), greater neutrophil numbers were observed in VIT-treated mice (Supplementary Figure S1O). This infection independent effect could indicate mild inflammatory or stress response resulting from treatment administration or FPN inhibition.

**Figure 2:**
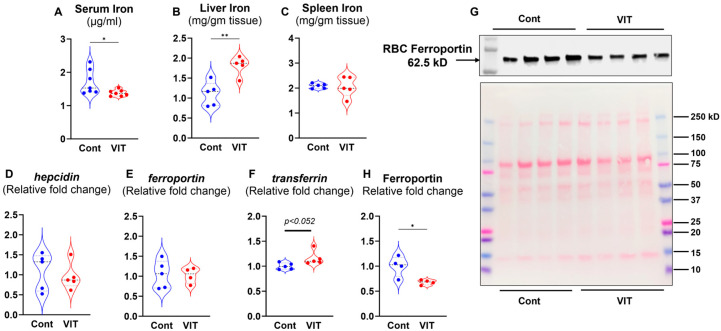
**Ferroportin inhibitor, VIT lowered circulating iron and enhanced liver iron.** WT mice (10-week-old males) were divided into two groups (*n* = 5/group); one group received 30 mg/kg bw of VIT and the control group received H_2_O, administered orally for 7 consecutive days). Mice were euthanized after the final dose. (**A**) Serum iron, (**B**) liver iron, (**C**) spleen iron. Hepatic mRNA expression of (**D**) *hepcidin*, (**E**) *ferroportin*, (**F**) *transferrin*. *18s* used as the housekeeping gene. (**G**) Immunoblot for Ferroportin in RBC membrane (*n* = 4/group). Each lane represents RBC membrane fraction isolated from a single mouse. Ponceau S staining is shown as loading control. (**H**) Densitometric analysis of immunoblot by ImageJ.JS. Data represented as mean ± SEM. * *p* < 0.05, ** *p* < 0.01.

Next, we assessed the extent to which pharmacologic inhibition of FPN affects the iron status of VIT-treated mice. Compared to the control group, VIT-treated mice showed reduced serum iron levels and elevated hepatic iron levels (Figure 2A), with no significant changes observed in splenic iron levels (Figure 2B,C). Although hepatic *hepcidin* and *Fpn* expression was comparable in both groups, a modest increase in *transferrin* expression was observed in VIT-treated mice (Figure 2D–F). Interestingly, VIT treatment led to a substantial reduction in FPN levels in RBC (Figure 2G,H), which is striking in indicating that VIT treatment differentially regulates FPN expression in hepatic tissue vs. RBCs (Figure 2E,G,H). In addition, serum levels of the liver injury marker alanine aminotransferase (ALT), the multi-organ damage marker aspartate aminotransferase (AST), total cholesterol, and pro-inflammatory markers such as serum lipocalin 2 (Lcn2) and serum amyloid A (SAA) were not significantly different between the VIT-treated and control groups (Supplementary Figure S1P–T). These results demonstrated oral VIT treatment effectively reduces serum iron and increases hepatic iron stores without causing systemic inflammation or liver injury in healthy mice.

### 3.3. Inhibition of FPN Aggravates the P. yoelii Infection

**Figure 3:**
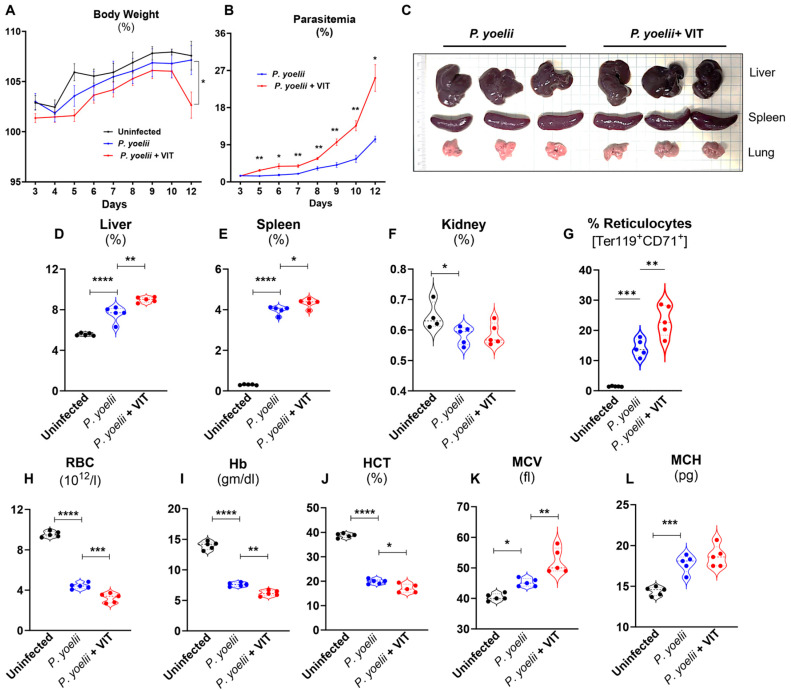
**VIT-treated mice showed greater *P. yoelii* infection.** Following infection with *P. yoelii*, WT mice (10-week-old males) were divided into two groups (*n* = 5/group): One group received 30 mg/kg bw of VIT, and the control group received H_2_O, administered orally 18 h *p.i.* The VIT-treated group received the same dosage of VIT on days 2, 4, 5, 6, 7, and 8 *p.i*., for a total of 7 doses; the control group received H_2_O at the same time points. The mice were euthanized on day 12 *p.i.* (**A**) % Body weight loss. (**B**) % Parasitemia. (**C**) Gross organ (liver, spleen and lung) pictures (**D**) % Liver weight, (**E**) % Spleen weight, (**F**) % Kidney weight. (**G**) Flow cytometric analysis of reticulocytes (Ter119^+^CD71^+^cells) in peripheral blood at day 12 *p.i.* Blood samples were analyzed for CBC. Results for: (**H**) RBC, (**I**) Hb, (**J**) HCT, (**K**) MCV and (**L**) MCH. Data represented as mean ± SEM. * *p* < 0.05, ** *p* < 0.01, *** *p* < 0.001 and **** *p* < 0.0001.

The interaction between hepcidin and FPN in iron regulation plays a critical role in severity and progression of *Plasmodium* infection. Hepcidin is upregulated during blood-stage parasitemia and facilitates the iron redistribution that regulates the disease’s severity [6]. VIT-2763 (Vamifeport), a novel oral FPN inhibitor has demonstrated promising therapeutic potential in both β-thalassemia [14] and sickle cell anemia [15]. Given that anemic conditions are exceedingly prevalent in malaria [29,30] and *Plasmodium* infection causes iron dysmetabolism [6], we examined the effects of VIT during progression of *P. yoelii* infection. We observed VIT-treated infected mice started to lose body weight 10 days *p.i.* (Figure 3A). Additionally, The VIT-treated mice showed an increase in parasitemia from day 5 *p.i.* compared to vehicle-treated *P. yoelii* infected mice (Figure 3B). On day 12 *p.i*, gross organ weights showed a significant increase in liver and spleen weights, with no changes in kidney weight, in the VIT-treated infected group compared to the vehicle-treated *P. yoelii* infected mice. Moreover, the lungs of the VIT-treated infected group appeared visibly darker compared to those of the vehicle treated group, suggesting underlying pathological changes in lung tissue (Figure 3C–F). The hematological parameters showed that VIT-treated infected mice suffered from severe anemia as evident by their increased reticulocytes (i.e., immature RBC, Ter119^+^ CD71^+^) (Figure 3G) and lower RBC numbers, Hb level and HCT compared to their untreated counterparts (Figure 3H–J). Interestingly, there was a significant increase in MCV value in VIT-treated group but no significant difference in MCH value (Figure 3K–L). These results showed that VIT-treated *P. yoelii* infected mice suffered from severe anemia.

### 3.4. VIT-Treated P. yoelii Infected Mice Showed Heightened Inflammatory Responses

**Figure 4:**
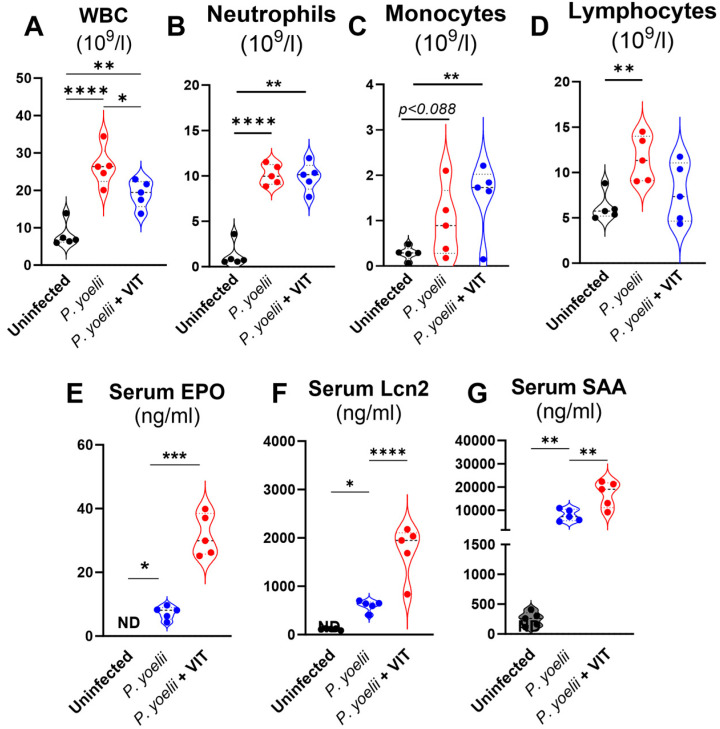
**VIT treatment heightened inflammatory responses in *P. yoelii* infected mice.** Following infection with *P. yoelii*, WT mice (10-week-old males) were divided into two groups (*n* = 5/group): One group received 30 mg/kg bw of VIT, and the control group received H_2_O, administered orally 18 h *p.i.* The VIT-treated group received the same dosage of VIT on days 2, 4, 5, 6, 7, and 8 *p.i*., for a total of 7 doses; the control group received H_2_O at the same time points. The mice were euthanized on day 12 *p.i.* CBC results for: (**A**) White blood cells (WBC), (**B**) Neutrophils (**C**) Monocytes and (**D**) Lymphocytes. Serum samples were analyzed for inflammatory cytokines, (**E**) EPO, (**F**) Lcn2 and (**G**) SAA. Data represented as mean ± SEM. * *p* < 0.05, ** *p* < 0.01, *** *p* < 0.001 and **** *p* < 0.0001.

*Plasmodium* infections trigger inflammatory responses that can disrupt normal immune regulation, resulting in the alteration of peripheral immune cells dynamics [31]. *Plasmodium* infection elevated circulatory WBCs, neutrophils, monocytes and lymphocytes numbers compared to the uninfected controls (Figure 4A–D). However, VIT treatment reduced the total WBC counts, while the numbers of neutrophils, monocytes and lymphocytes remained unchanged (Figure 4A–D). Anemia is an important clinical manifestation of malaria, resulting from both increased RBC lysis and impaired RBC production due to ineffective erythropoiesis [32]. In response to anemia, the host tries to compensate by increasing the production of erythropoietin (EPO), a hormone which promotes erythropoiesis [33,34,35] and stimulates the generation of reticulocytes, the immature red blood cells [36]. Compared to uninfected control mice, *P. yoelii* infection significantly increased the percentage of reticulocytes as we showed before (Figure 3G), with a more pronounced increase observed in VIT- treated mice (Figure 3G). Supporting these results, in control mice, the serum EPO levels were undetectable, while *P. yoelii* infection significantly enhanced the serum EPO levels (Figure 4E). Interestingly, VIT treatment further increased the serum EPO levels in *P. yoelii* infected mice, supporting the presence of severe anemia and dyserythropoesis in VIT-treated infected group, consistent with our earlier findings (Figure 3). Notably, acute inflammatory cytokines, such as serum Lipocalin 2 (Lcn2) and serum amyloid A (SAA) were significantly increased in the VIT-treated infected group compared to the vehicle-treated infected group (Figure 4F,G). These results indicated that inhibition of FPN aggregates the inflammatory response in *P. yoelii* infection.

### 3.5. VIT-Treated P. yoelii Infected Mice Showed Increased Hepatic Injury and Inflammation

**Figure 5:**
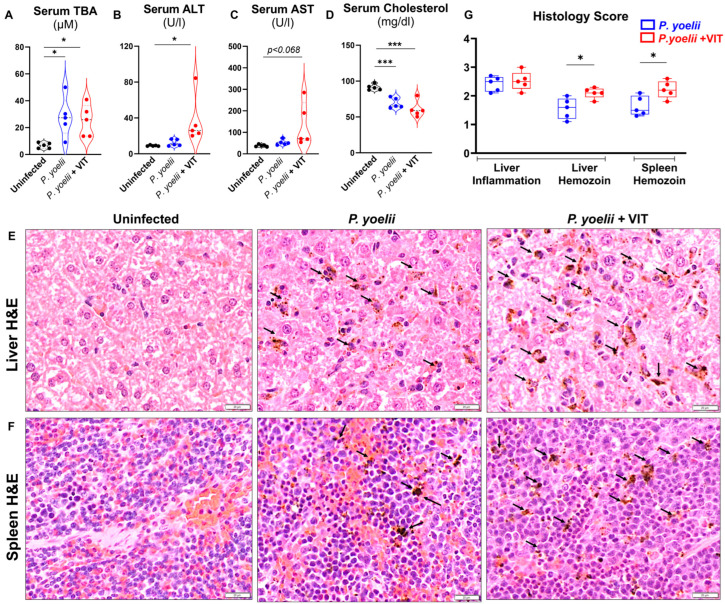
**VIT treatment aggravated inflammation in *P. yoelii* infected mice.** Following infection with *P. yoelii*, WT mice (10-week-old males) were divided into two groups (*n* = 5/group): One group received 30 mg/kg bw of VIT, and the control group received H_2_O, administered orally18 h *p.i.* The VIT-treated group received the same dosage of VIT on days 2, 4, 5, 6, 7, and 8 *p.i*., for a total of 7 doses; the control group received H_2_O at the same time points. The mice were euthanized on day 12 *p.i.* Serum samples were analyzed for serum (**A**) total bile acids (TBA), (**B**) alanine transaminase (ALT), (**C**) aspartate aminotransferase (AST) and (**D**) cholesterol. The liver and spleen sections were processed for H&E staining to assess histopathological changes. (**E**) Liver histology: bars are 20 μm (40× magnification). (**F**) Spleen histology: bars are 20 μm (40× magnification). Parasitized red blood cells and hemozoin pigments are marked with black arrows. (**G**) Histological score based on liver inflammation and hemozoin deposition in liver and spleen in *P. yoelii* infected mice with and without VIT treatment. Data represented as mean ± SEM. * *p* < 0.05 and *** *p* < 0.001.

Hepatic dysfunction and cholestasis are common features of severe malaria [37,38,39]. The liver cholestasis marker, serum total bile acids (TBA) were elevated following *P. yoelii* infection and were comparable in both infected groups with and without VIT treatment (Figure 5A). In contrast, serum ALT and AST were moderately higher in VIT-treated *P. yoelii*-infected mice compared to their untreated counterparts (Figure 5B,C). Additionally, serum cholesterol levels were significantly reduced in all *P. yoelii*-infected mice, suggesting greater liver dysfunction in these mice on day 12 *p.i.* (Figure 5D). Histological analysis of H&E-stained liver and spleen sections revealed significant accumulation of malarial parasite pigment, hemozoin, in both *P. yoelii* infected groups, with a more pronounced deposition observed in the VIT-treated mice (Figure 5E,G). Such pathology in VIT-treated infected mice corroborated higher serum ALT (Figure 4B). These findings suggested that malarial hepatitis is more prominent in VIT-treated *P. yoelii*-infected mice. Furthermore, compared to infected mice, histological analysis of spleen sections from VIT-treated infected mice showed notable differences, including a more pronounced loss of central germinal structure, and absence of red pulp and white pulp regions (Figure 5F,G). The parasitized RBC deposition (black arrows) was more prominent in spleen sections from VIT-treated *P. yoelii* infected mice.

### 3.6. VIT Treatment Increased Hepatic Fpn Expression and Suppressed RBC-FPN in P. yoelii-Infected Mice

**Figure 6:**
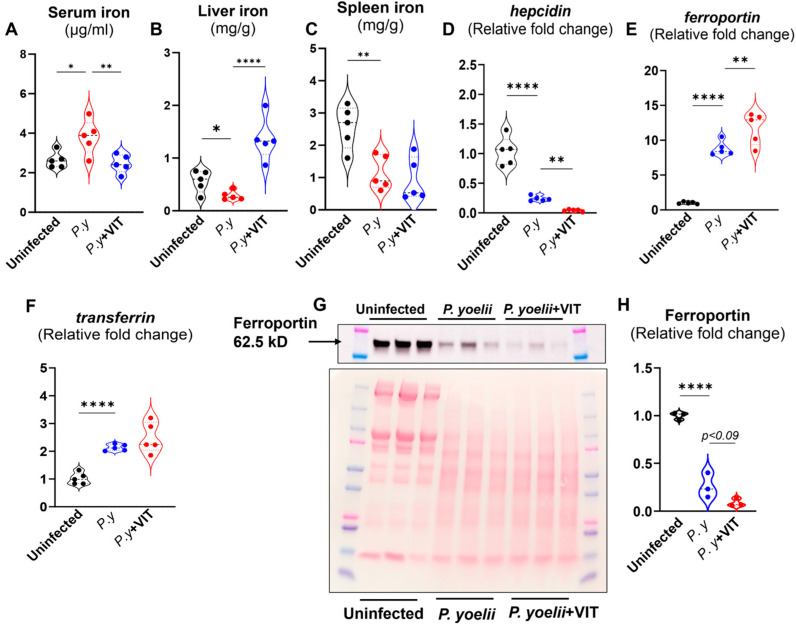
**VIT treatment upregulated hepatic *Fpn* expression and suppressed RBC-FPN protein levels in *P. yoelii* infected mice.** Following infection with *P. yoelii*, WT mice (10-week-old males) were divided into two groups (*n* = 5/group): One group received 30 mg/kg bw of VIT, and the control group received H_2_O, administered orally18 h *p.i.* The VIT-treated group received the same dosage of VIT on days 2, 4, 5, 6, 7, and 8 *p.i*., for a total of 7 doses; the control group received H_2_O at the same time points. The mice were euthanized on day 12 *p.i.* (**A**) Serum Iron, (**B**) liver iron, (**C**) spleen iron. Hepatic mRNA expression of (**D**) *hepcidin*, (**E**) *ferroportin*, (**F**) *transferrin. 18s* used as the housekeeping gene. (**G**) Immunoblot for Ferroportin in RBC membrane (*n* = 3/group). Each lane represents RBC membrane fraction isolated from a single mouse. Ponceau S staining is shown as loading control. (**H**) Densitometric analysis of immunoblot by ImageJ.JS. Data represented as mean ± SEM. * *p* < 0.05, ** *p* < 0.01, and **** *p* < 0.0001.

To investigate the impact of VIT treatment on *P. yoelii* infection, we first measured serum iron levels in the vehicle and VIT-treated *P. yoelii* infected mice. We observed that in vehicle -treated *P. yoelii* infected mice the serum iron levels were elevated, whereas VIT treatment significantly reduced serum iron levels (Figure 6A). Interestingly, liver iron levels were reduced following *P. yoelii* infection but significantly increased upon VIT treatment, indicating enhanced iron retention in the liver (Figure 6B). Conversely, splenic iron levels were significantly decreased in the infected mice and remained unchanged with VIT treatment (Figure 6C). Next the hepatic *hepcidin* expression, which generally downregulated in 12 days *p.i.*, was further suppressed by VIT treatment, likely contributing to the increased hepatic iron accumulation (Figure 6D). Consistently, hepatic *Fpn* expression was elevated in response to *P. yoelii* infection and further upregulated with VIT treatment (Figure 6E). *Transferrin* expression was upregulated in both infected groups and remained unchanged by VIT treatment (Figure 6F). Given the importance of the RBC-FPN in malaria pathogenesis [11,40,41], we next determined RBC-FPN expression. Immunoblot analysis revealed that RBC-FPN levels were further reduced in VIT-treated infected mice compared to untreated infected group (Figure 6G,H). This suppression of RBC-FPN may impair iron export from RBCs, increasing their susceptibility to hemolysis and creating intraerythrocytic conditions that favor *Plasmodium* development.

## 4. Discussion

Iron is an essential nutrient for both the host and the *Plasmodium* parasites, and its homeostasis is tightly regulated by the hepcidin-FPN axis. Since *Plasmodium* spp. depends on iron for survival and replication within RBCs, this regulatory pathway is closely linked to disease progression and severity [4,6]. The mRNA of *Fpn*, primarily expressed in enterocytes, hepatocytes, and erythroid precursors, contains a 5′ iron-responsive element (IRE) that regulates its translation in response to intracellular iron levels [42,43]. Iron regulatory proteins provide an additional mechanism of post-transcriptional regulation of FPN by binding to the 5′ IRE site and inhibiting *Fpn* translation under low-iron conditions [44]. At the post-translational level, hepcidin, a liver-derived peptide hormone, binds to ferroportin and induces its internalization and degradation, thereby reducing cellular iron export. Although this response may help restrict iron availability to the parasite, it can also impair erythropoiesis and contribute to malarial anemia, a common and severe complication of malaria [6,9,10,11].

Our study demonstrates that, during *P. yoelii* infection, there are significant temporal changes in the expression of key iron-regulatory proteins. Specifically, we observed a downregulation of hepatic *hepcidin* and upregulation of *Fpn* and *transferrin* on day 12 *p.i*, coinciding with increased erythropoietic drive and anemia. These results suggest a shift toward iron mobilization in the later stage of infection. This may reflect a host attempt to support erythropoiesis and counter anemia. Several studies have shown that *hepcidin* expression is typically upregulated during the early phase of *Plasmodium* infection due to inflammation-driven signals, including IL-6, as part of the host’s innate immune response to limit iron availability to the parasite. However, as the infection progresses and anemia develops, erythropoietic drive increases, leading to elevated EPO and subsequent production of erythroferrone (ERFE), which suppresses hepcidin [28,45,46]. In line with these findings, in our study, on day 12 *p.i.*, both VIT-treated and untreated groups exhibited elevated serum EPO levels, with a more marked increase in the VIT-treated group, suggesting a compensatory erythropoietic response to heightened malaria severity and anemia. Together with the reduction in *hepcidin*, these results point to enhanced erythropoietic drive and iron mobilization.

VIT, a pharmacological ferroportin inhibitor, is currently undergoing clinical development, and has demonstrated good tolerability in healthy volunteers [19]. In mouse models of sickle cell disease and β-thalassemia, VIT improved hematologic parameters by reducing hemolysis, correcting anemia, and restoring iron homeostasis [15,17,18]. These effects were largely attributed to restricted iron release and reduced iron overload. In our study, in uninfected control mice, VIT treatment selectively reduced RBC-FPN levels without affecting hepatic *FPN*, underscoring a pronounced tissue-specific effect of VIT. Given these observations, we sought to investigate the impact of VIT treatment in a mouse model of malaria driven by the rodent-specific *P. yoelii*. Interestingly, VIT treatment aggravates the *P. yoelii* infection. Indeed, VIT treatment further reduced FPN levels in RBCs and exaggerated disease pathology. However, the hepatic *FPN* expression was instead upregulated VIT-treated *P. yoelii*-infected mice. Such contrasting results suggest that VIT may be exerting a site-specific effect, whereby FPN expression of RBC was more liable for inhibition. It is also plausible that there are compensatory mechanisms in the liver that sustain FPN expression, which prevents VIT from inhibiting hepatic FPN expression. These contrasting results highlight the context-dependent impact of ferroportin inhibition; while beneficial in non-infectious anemias, restricting iron release during malaria may enhance parasite growth and worsen outcomes by increasing intracellular iron availability.

The impact of RBC-FPN in *Plasmodium* infection has been demonstrated in different studies [11,40,41]. Our findings revealed that FPN protein levels in RBC were reduced upon *P. yoelii* infection. Moreover, VIT treatment exacerbated the severity of *Plasmodium* infection, which was associated with a more pronounced reduction in RBC-FPN levels. These results align with previous studies demonstrating that deletion of the *Fpn* gene in RBC in mice leads to intracellular iron accumulation, increased oxidative stress, hemolytic anemia, and greater malaria mortality, highlighting the detrimental consequences of iron overload during infection [11]. The high levels of iron and reactive oxygen species (ROS) generated during malaria infection can drive iron-dependent lipid peroxidation, a central feature of ferroptosis. Ferroptosis may influence malaria pathogenesis by contributing to parasite eradication or promoting the destruction of infected RBCs and hepatocytes. In response, the parasites counteract this ferroptosis-like threat by converting toxic heme into inert hemozoin and altering the host and parasite membrane lipid composition [47,48].

The consequences of hemozoin accumulation are complex and far-reaching. Hemozoin, composed of aggregated heme along with lipids, lipid derivatives, and proteins of both parasite and host origin, has been implicated in promoting oxidative stress. This includes the generation of ROS, which can trigger lipid peroxidation. RBCs are particularly susceptible to ROS-induced lipid peroxidation due to their high membrane polyunsaturated fatty acid content and lack of organelles for detoxification. Key byproducts of this process, 4-hydroxynonenal (4-HNE and malondialdehyde), are -NH2 group reactive aldehydes known to modify proteins, nucleic acids, and lipids, thereby impairing the function of essential biomolecules. Such damage can contribute to anemia by disrupting erythropoiesis and compromising RBC membrane integrity. Additionally, 4-HNE can impair immune cell signaling and survival, potentially playing a role in the immunosuppression observed in severe malaria [49,50,51,52]. Beyond hematologic and immune effects, hemozoin has also been shown to be taken up by neurons and astrocytes, where it disrupts the regulation of pro-apoptotic proteins, ultimately inducing apoptosis in both cell types. These findings suggest that hemozoin may contribute directly to the central nervous system dysfunction seen in cerebral malaria [53]. Here, histological analysis of liver and spleen sections revealed significant accumulation of hemozoin, in both *P. yoelii* infected groups, with a more pronounced deposition observed in the VIT-treated mice.

In humans, a genetic variant of ferroportin called Q248H (glutamine to histidine at position 248) makes the protein resistant to degradation by hepcidin [54]. The impact of the FPN Q248H mutation on malaria remains controversial, with studies reporting conflicting outcomes ranging from protection against malaria, to protection against anemia but not malaria, to no protection against either condition [11,13,46,54]. Nonetheless, the Q248H mutation may confer some protection against malaria by reducing parasite burden and mitigating disease severity [11]. Moreover, our study revealed an increase in hepatic *Fpn* expression following VIT treatment, in the context of reduced hepatic *hepcidin* levels. Under normal physiological conditions, the ferroportin expression is tightly regulated by hepcidin: low hepcidin levels typically permit stabilization and surface expression of ferroportin, while high hepcidin leads to its degradation. The observed upregulation of hepatic *Fpn* despite VIT-mediated inhibition suggests a compensatory transcriptional response by the liver aimed at preserving systemic iron to support RBC production, particularly under *Plasmodium* infection where iron availability critically influences disease severity.

Furthermore, the concurrent elevation of hepatic *transferrin* levels in both infected and VIT-treated mice further supports the notion of increased iron mobilization and delivery to developing erythroid precursors. Transferrin, the primary iron transport protein in circulation, reflects systemic iron demand and its saturation status is a key determinant of iron delivery to tissues [55]. However, this adaptive response could have detrimental consequences in the context of malaria; by increasing the pool of bioavailable iron, particularly in the form of transferrin-bound iron, the host may inadvertently facilitate greater access to iron by the parasite. This could enhance intraerythrocytic parasite proliferation and exacerbate disease severity, including worsening of anemia. These findings highlight the delicate balance between host iron regulation and pathogen exploitation, and underscore the complexities associated with pharmacologically targeting the hepcidin-FPN axis in context of *Plasmodium* infection.

Given the geographic overlap between malaria-endemic regions and the high prevalence of β-thalassemia and sickle cell disease, further investigations of FPN modulation in human RBCs infected with anthropophilic species such as *P. falciparum* and *P. vivax* are needed to better inform the use of iron-modulating therapies. While these treatments may benefit patients with hemoglobinopathies, they could pose risks in populations also burdened by malaria. Manipulating the pathway in the opposite direction such as transiently suppressing hepcidin or enhancing ferroportin activity may represent a potential therapeutic strategy to alleviate malaria-associated pathology. However, this approach would need to be carefully balanced, as excessive iron availability could also enhance parasite replication. These findings underscore the need for individualized, disease-specific strategies when targeting iron metabolism, balancing the management of chronic anemia with the risks of infectious disease.

## 5. Conclusions

Our findings emphasize the critical role of RBC-FPN in regulating *Plasmodium* infection and disease severity. While the host’s iron-sequestering response may initially act to limit parasite proliferation; however, iron restriction impairs erythropoiesis and may contribute to disease severity. Therapeutic modulation of the hepcidin-FPN axis must therefore strike a delicate balance between limiting iron availability to the parasite and preserving sufficient iron for effective erythropoiesis and immune functions. Future studies should be considered, especially in populations burdened by both iron dysregulation and infectious diseases such as malaria.

## Data Availability

The original contributions presented in this study are included in the article/Supplementary Material. Further inquiries can be directed to the corresponding author.

## Supplementary Materials

The following supporting information can be downloaded at: https://www.mdpi.com/article/10.3390/microorganisms13081859/s1, Figure S1: VIT treatment did not induce inflammation in WT mice. WT mice (10-week-old males) were divided into two groups (*n* = 5/group); one group received 30 mg/kg bw of VIT and the control group received H2O, administered orally for 7 consecutive days. Mice were euthanized after the final dose. (A) % Body weight. (B) % Spleen weight, (C) % Liver weight, (D) % Kidney weight. Blood samples were collected (EDTA tubes) for CBC analysis. Results for: (E) Red blood cells (RBC); (F) Hemoglobin (Hb); (G) Hematocrit (HCT, the volume percentage of RBC in blood), (H) Mean Corpuscular Volume (MCV), (I) Mean Corpuscular Hemoglobin (MCH), (J) Mean Corpuscular Hemoglobin Concentration (MCHC), and (K) Platelets (PLT), (L) White blood cells (WBC), (M) Lymphocytes, (N) Monocytes and (O) Neutrophils. Serum samples were analyzed for (P) cholesterol (Q) alanine transaminase (ALT), (R) aspartate aminotransferase (AST), cytokines including (S) Lipocalin 2 (Lcn2), (T) serum amyloid A (SAA). Data represented as mean ± SEM. * *p* < 0.05.

## Abbreviations

The following abbreviations are used in this manuscript:

ALT	Alanine aminotransferase
AST	Aspartate transaminase
CBC	Complete blood count
EPO	Erythropoietin
FPN	Ferroportin
Hb	Hemoglobin
HCT	Hematocrit
*i.p.*	Intra-peritoneally
IRE	Iron-response element
Lcn2	Lipocalin 2
MCH	Mean corpuscular hemoglobin
MCHC	Mean corpuscular hemoglobin concentration
MCV	Mean corpuscular volume
*p.i.*	Post-infection
*P. yoelii*	*Plasmodium yoelii*
RBC	Red blood cell
RBC-FPN	Red blood cell Ferroportin
SAA	Serum amyloid A
TBA	Total bile acids
VIT	VIT-2763
WBC	White blood cell
