# Supplementary material for: Pharmacologic Inhibition of Erythrocyte Ferroportin Expression Exacerbates Plasmodium Infection
Source: Microorganisms. 2025 Aug 8;13(8):1859. doi: 10.3390/microorganisms13081859 (PMC12388592; doi:10.3390/microorganisms13081859)
Supplement: Supplementary file 1 [file microorganisms-13-01859-s001.zip › microorganisms-3717388-supplementary.pdf]

Supplementary Figure 1

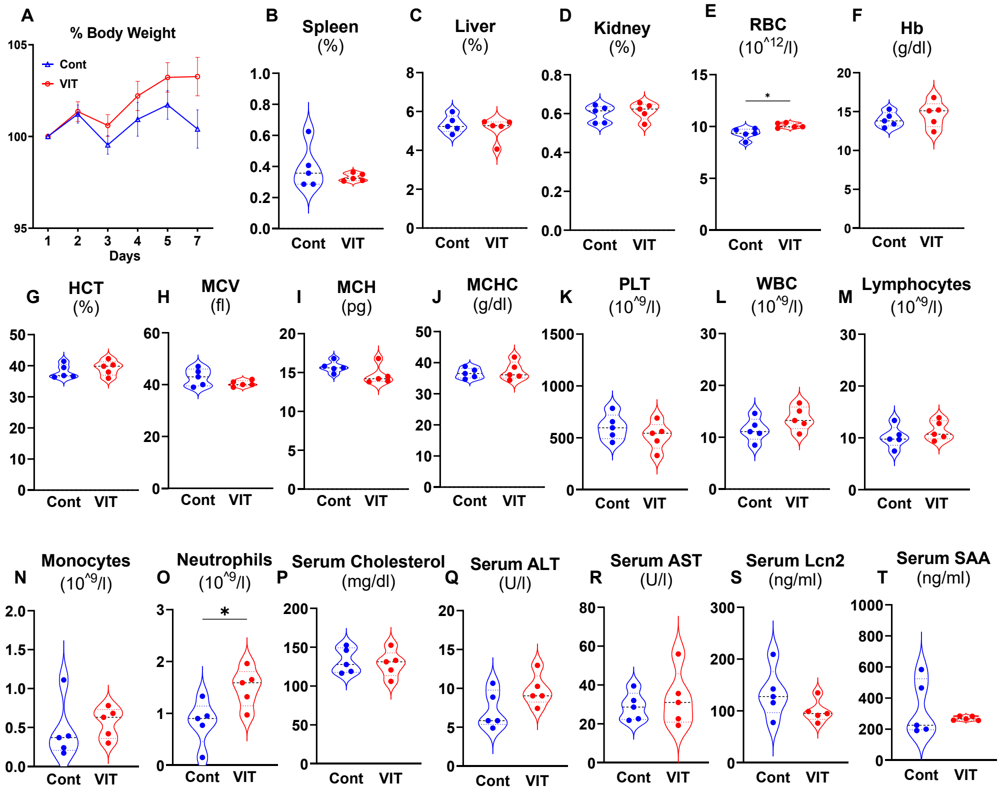

Supplementary Figure S1: VIT treatment did not induce inflammation in WT mice. WT mice (10-week-old males) were divided into two groups (n=5/group); one group received 30 mg/kg bw of VIT and the control group received H_2O, administered orally for 7 consecutive days. Mice were euthanized after the final dose. **(A)** % Body weight. **(B)** % Spleen weight, **(C)** % Liver weight, **(D)** % Kidney weight. Blood samples were collected (EDTA tubes) for CBC analysis. Results for: **(E)** Red blood cells (RBC); **(F)** Hemoglobin (Hb); **(G)** Hematocrit (HCT, the volume percentage of RBC in blood), **(H)** Mean Corpuscular Volume (MCV), **(I)** Mean Corpuscular Hemoglobin (MCH), **(J)** Mean Corpuscular Hemoglobin Concentration (MCHC), and **(K)** Platelets (PLT), **(L)** White blood cells (WBC), **(M)** Lymphocytes, **(N)** Monocytes and **(O)** Neutrophils. Serum samples were analyzed for **(P)** cholesterol **(Q)** alanine transaminase (ALT), **(R)** aspartate aminotransferase (AST), cytokines including **(S)** Lipocalin 2 (Lcn2), **(T)** serum amyloid A (SAA). Data represented as mean ± SEM. $^*p<0.05$.
